# Pericardial mass: A rare form of cardiac actinomycosis case report

**DOI:** 10.1016/j.amsu.2022.103854

**Published:** 2022-05-24

**Authors:** Abdulhadi Almutairi, Ahsan Cheema, Amani Joudeh, Ayed Alqarni, Suha Albadr, Manal Alnaimi

**Affiliations:** a Department of Surgery, King Fahad Specialist Hospital, Dammam, 34423, Saudi Arabia; b Department of Pathology, King Fahad Specialist Hospital, Dammam, 34423, Saudi Arabia; c Department of Radiology, King Fahad Specialist Hospital, Dammam, 34423, Saudi Arabia

**Keywords:** Pericardial, Actinomycosis, Surgery, Diagnosis, Tumor, Case report

## Abstract

Actinomycosis is a rare chronic infection caused by a group of anaerobic Gram-positive bacteria which inhabits commonly the oral cavity, colon, and genitourinary tract. Actinomycosis of the thorax is the third most common form. Pericardial actinomycosis is an extremely rare condition. Actinomycosis is characterized by its tendency to mimic malignancy as it can invade surrounding tissue and form a mass. Multiple manifestations should be noted by physicians as a result of the large variety of symptoms and the involvement of multiple organ systems. With proper treatment, it has a good prognosis. We describe a patient with an unusual clinical form of cardiac actinomycosis presenting as an isolated pericardial mass resembling a malignant tumor.

## Highlights

• Actinomycotic infection is rare but treatable disease.
• Pericardial involvement in actinomycotic infection is rare.
• Cardiac actinomycosis is usually secondary to contiguous spread from a pulmonary focus.
• Prognosis is excellent if the infection is diagnosed early and treated appropriately.
• This is a rare case of isolated pericardial actinomycosis mimicking malignancy treated successfully with surgery and long term penicillin therapy.

## 1 Introduction

Actinomyces species may cause human infections through multi-system involvement. Clinical presentation varies widely, and the likelihood of a successful outcome is higher when prompt diagnosis and treatment are implemented. Cardiac actinomycosis has frequently presented as pericardial effusion, pericarditis, and rheumatic fever. Contagious spread from adjacent pulmonary disease has also been reported. Even though this patient presents with a productive cough, weight loss, and declining general health, the mass on the pericardium simulates the appearance of malignancy. It was successfully removed surgically and treated with prolonged antibiotic therapy. In addition to weight gain, the patient experienced an improved cough and was followed for three years. This case report has been reported inline with SCARE criteria [1].

## 2 Case presentation

While being evaluated for left shoulder pain, a 77-year-old man with no medical history had an abnormal chest X-ray. The patient was referred from his primary care clinic to our outpatient thoracic clinic for further evaluation. During the past two months, he complained of deteriorating health, loss of appetite, weight loss of 7 kg, and worsening pain in his left shoulder. Additionally, he developed a cough that caused yellow sputum and mild exertional dyspnea. Since he was 27 years old, he has smoked a pack of cigarettes every day.

The patient was not experiencing shortness of breath at the time of presentation. He had an oral temperature of 37.7 C, a pulse rate of 85 beats per minute, and a respiratory rate of 18 beats per minute. No clubbing was present. The oropharyngeal examination revealed poor teeth with extensive caries. There were no findings of cervical lymphadenopathy or dilated jugular veins. Cardiovascular and respiratory examinations were normal.

**Fig. 1:**
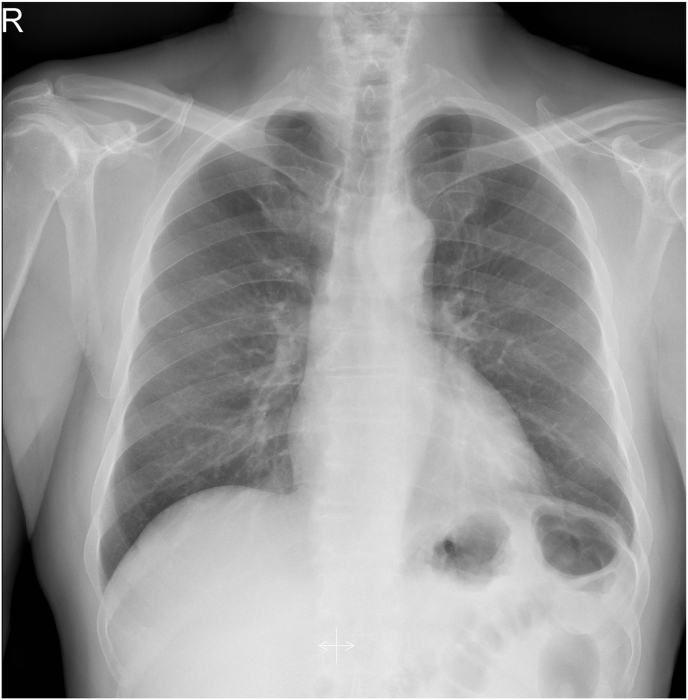
Chest radiograph showing a left lower thoracic opacity that obscure the left heart border.

Laboratory findings indicated microcytic anemia (hemoglobin concentration of 11 g/dl), white blood cell count of 11 x 109/l, 60% neutrophils, 30% lymphocytes, 8% monocytes, and 2% basophils. The electrocardiogram showed no abnormalities, and the voltage was normal. A Gram stain of the patient's sputum revealed no microorganisms. Additionally, Ziehl-Neelsen staining was negative. An x-ray of the chest revealed opacity in the lower left chest, obscuring the left side of the heart Fig. 1.

**Fig. 2:**
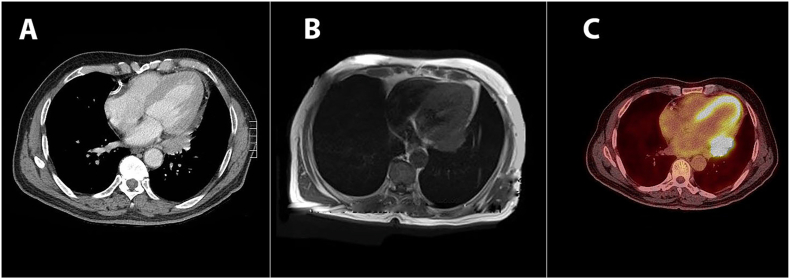
Chest computed tomography (CT) showing posteriorly located pericardial mass (A). Cardiac magnetic resonant imaging (MRI) confirming the location of the mass (B). Positron emission tomography (PET) scan showing high uptake in the pericardial mass (SUV max 7) (C).

**Fig. 3:**
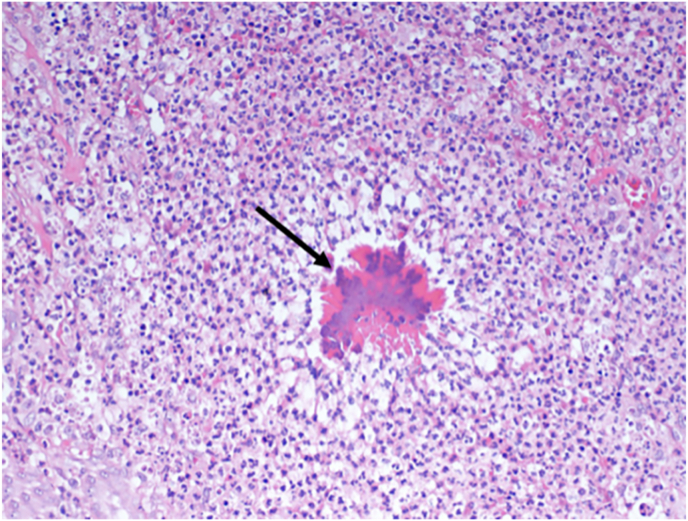
High power view showing a colony of actinomyces species featuring filaments radiating from the center. (H&E stain, 200X).

Computed tomography of the chest revealed a small pericardial mass adjacent to the left lung Fig. 2 (A). The study did not identify any other intrathoracic abnormalities. The mass was further characterized by cardiac magnetic resonance imaging (MRI) study Fig (2B). Transthoracic echocardiography revealed pericardial thickening without pericardial effusion. A positron emission tomography (PET) scan showed an FGD-intensive pericardial mass with a standard uptake value (SUV^max^) of 7 Fig. 2 (C). Pericardial malignancy is suspected in light of these findings. Different diagnoses include primary pericardial tumors, metastases, granulomas, and inflammatory masses. We, therefore, decided to perform a thoracoscopic mass excision under general anaesthesia. The chest cavity looks normal during thoracoscopic surgery, with no pleural or parenchymal disease. A 3 × 2 cm hard pericardial mass was found and surgically removed. The patient had a smooth recovery and returned to their routine postoperatively. In histopathology, filamentous rods corresponding to actinomycete colonies were detected Fig. 3. Penicillin G was given intravenously to the patient at a dose of 400,000 units per kilogram of body weight per day for a month. In addition, oral penicillin was given to the patient for a further six months. A repeat computed tomography of the chest did not reveal any recurrence or residual disease. At the 3- year follow-up, the patient was doing well with no other symptoms.

### 2.1 Clinical discussion

Actinomycosis is a rare infection caused by filamentous, Gram-positive, non-acid- fast, anaerobic bacteria, Actinomyces species. These species belong to the natural flora on the mucosal surfaces of the oral, gastrointestinal and genitourinary tracts and can cause endogenous infections. Humans are the natural host, and there is no evidence of human-to-human transmission of the disease [2]. Mucosal injury at almost any site precedes infection, leading to different clinical manifestations. At least 30 species have been isolated, of which the most common infection-causing microorganisms are A. israelii, A. naeslundii, A. odonolyticus, A. viscosus, A. meyeri, and A. gerencseriae [3]. It is characterized by an extensive, suppurative, granulomatous inflammation with multiple abscesses and sinus ducts that may emit sulfur particles. Although actinomycosis is not considered an opportunistic infection, it has been described in patients with HIV [4] and leukemia [5] and patients with other causes of immunodeficiency. However, most patients have no underlying disease or immunodeficiency [6].

In particular, because of their rare and nonspecific clinical symptoms, actinomycosis is a fascinating clinical condition that can easily be confused with other diseases, including tuberculosis and malignancies [[7], [8]]. Actinomycotic infections were classified by anatomical site; cervical and facial disease was most common (55%), followed by abdominopelvic (20%), thoracic (15%), and mixed organ involvement (10%), including skin, brain, pericardium, and extremities [8].

Diagnosis of actinomycosis is often hindered by the ability to isolate and culture the organism. It must be cultivated under strictly anaerobic conditions. Direct isolation of microorganisms from clinical specimens is essential for definitive diagnosis. The high failure rate (greater than 50%) of isolation is due to previous antibiotics, suboptimal methods, and microbial overgrowth. Therefore, prolonged anaerobic culture in a selective agar medium for up to 3 weeks is required [9].

Most cases of actinomycosis reported in the literature are diagnosed by histopathology. Shinagawa and colleagues [10] reported that the pericardial biopsy did not provide convincing evidence of pericardial actinomycosis, but the culture of pericardial effusion showed positive growth. More recently, molecular techniques such as 16s ribosomal RNA sequencing have aided in identifying, and matrix-assisted laser desorption time-of-flight analysis promises faster and more accurate identification of actinomycetes in the future.

Actinomycosis rarely affects the heart. Kasper and Pinner [11] and Cornell and Shookhoff [12] collected the most extensive case series of cardiovascular actinomycosis, reporting an incidence of less than 2%. Although pericardial actinomycosis usually results from the continuous spread of thoracic actinomycosis, it may occur after primary myocardial or endocardial infection, hematogenous or transdiaphragmatic spread [13]. The typical presentation of pericardial actinomycosis is purulent pericarditis or pericardial effusion, which can progress to cardiac tamponade or constrictive pericarditis. Cardiac actinomycosis can also mimic rheumatic fever or manifest as actinomycete embolism [10,11]. Fife et al. [14] reviewed 19 cases of pericardial actinomycosis. Cardiac tamponade occurred in 10 patients (53%). A primary focus of chest infection was found in 15 patients (79%).

Janoskuti et al. [15] published a case of a pericardial mass similar to our case but with infectious pulmonary actinomycosis.

Actinomycosis is usually amenable to antibiotic therapy [16], and in the early stages, the prognosis is excellent. Local variants are rare, and surgical drainage or excision and antibiotic treatment can speed recovery. Severe hemoptysis, failure of medical therapy, or diagnostic uncertainty may also require surgery [17]. Treatment time is sometimes extended to 12 months. Several antibiotics, such as benzylpenicillin, cotrimoxazole, trimethoprim, and the amoxicillin-clavulanate combination, have been used and found to be effective [18]. The use of many antibiotics, the lack of drug resistance, and the advent of adjuvant methods such as percutaneous drainage and surgical resection have greatly improved the prognosis of actinomycosis. However, studies on drug therapy for cardiac-involving actinomycosis are rare.

## 3 Conclusion

Actinomycotic infection is a rare but treatable disease. Cardiac involvement is often accompanied by pericardial effusion or pericarditis. For the treatment of this infection, long-term antibiotics are necessary, while surgery has limited indications. This case report contributes to our current understanding of how to treat actinomycotic infections due to its unique clinical presentation, treatment modality, and long-term follow-up.

## Ethical approval

Granted from institutional board review (IRB).

## Sources of funding

None.

## Author contributions

Abdulhadi Almutairi performed the surgery and wrote the manuscript.

Ahsan Cheema performed the surgery and collected data.

Amani Joudeh reported the histology slides.

Ayed Algarni reviews the slides and wrote the abstract.

Suha Albadr reported radiological images.

Manal Alnaimi wrote the discussion and reviewed the manuscript.

## Registration of research studies

1. Name of the registry: research registry.

2. Unique Identifying number or registration ID: NA.

3. Hyperlink to your specific registration (must be publicly accessible and will be checked):

https://researchregistry.knack.com/research-registry#user-researchregistry/

## Guarantor

Abdulhadi Almutairi.

## Consent

Written informed consent was obtained from the patient for publication of this case report and accompanying images. A copy of the written consent is available for review by the Editor-in-Chief of this journal on request.

## Provenance and peer review

Not commissioned, externally peer-reviewed.

## Declaration of competing interest

None.
